# A case of idiopathic myointimal hyperplasia of the mesenteric veins presenting with small bowel obstruction

**DOI:** 10.1186/s40792-020-01100-8

**Published:** 2021-01-13

**Authors:** Kohei Yamada, Masatsugu Hiraki, Toshiya Tanaka, Daisuke Mori, Futoshi Tanaka, Tatsuya Manabe, Hitoshi Aibe, Kenji Kitahara, Hirokazu Noshiro

**Affiliations:** 1 Department of Surgery, Saga Medical Centre Koseikan, 400 Kasemachi, Nakabaru, Saga, 840-8571 Japan; 2 Department of Pathology, Saga Medical Centre Koseikan, 400 Kasemachi, Nakabaru, Saga, 840-8571 Japan; 3 Department of Radiology, Saga Medical Centre Koseikan, 400 Kasemachi, Nakabaru, Saga, 840-8571 Japan; 4 Department of Surgery, Faculty of Medicine, Saga University, 5-1-1 Nabeshima, Saga, 849-8501 Japan

**Keywords:** Idiopathic myointimal hyperplasia of mesenteric veins, Inflammatory bowel disease, Ileum, Bowel obstruction, Enterocolic lymphocytic phlebitis, Mesenteric inflammatory veno-occlusive disease

## Abstract

**Background:**

Idiopathic myointimal hyperplasia of the mesenteric veins (IMHMV) is a rare ischemic bowel disease with venous occlusion resulting from the proliferation of smooth muscles in the venous intima. In most patients, the disease affects rectosigmoid colon and causes persistent abdominal pain and hematochezia, which is similar to inflammatory bowel disease (IBD). In addition, it is difficult to make a precise diagnosis of IMHMV without surgery.

**Case presentation:**

An 81-year-old woman was admitted to our hospital with mild abdominal pain, nausea and vomiting. Repeated adhesive ileus was suspected due to the previous open and laparoscopic surgeries. Surgery was planned to treat small bowel obstruction. Intraoperatively no adhesive lesions were observed. However, a mass lesion was seen at the terminal ileum, which was suspected to have caused her bowel obstruction. Partial resection of the small intestine was performed. Macroscopic and histopathological examinations of the excised specimen showed circumferential ulceration with scarring, a thickened venous wall with active inflammation, and fibrotic changes that consequently produced stenosis and obstruction of the venous lumen in the subserosa. Additionally, Elastica van Gieson staining demonstrated thickening of the venous intima. The final diagnosis was IMHMV. At two years and 8 months after the operation, the patient was well without any additional medication.

**Conclusion:**

IMHMV of the small intestine is rare. We described a case of IMHMV that was associated with ileus.

## Background

Idiopathic myointimal hyperplasia of the mesenteric veins (IMHMV) is a rare ischemic bowel disease without thrombus and with venous occlusion resulting from the proliferation of smooth muscle in the venous intima [1]. In most of the patients, the disease affects the rectosigmoid colon and provides persistent abdominal pain and hematochezia [1]. IMHMV has therefore often been misdiagnosed as an inflammatory bowel disease (IBD), and it is difficult to diagnose without surgery [2]. We herein describe the case of a patient with small intestinal obstruction due to IMHMV.

## Case presentation

**Fig. 1:**
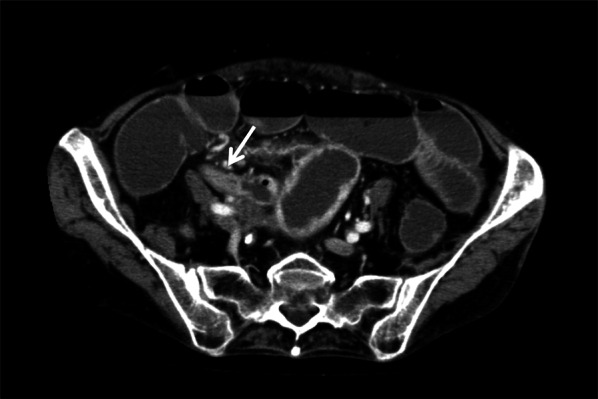
Computed tomography demonstrates that the whole bowel is distended, with an air-fluid level in the small intestine. Wall thickening and stenosis of the terminal ileum (white arrow) are found

**Fig. 2:**
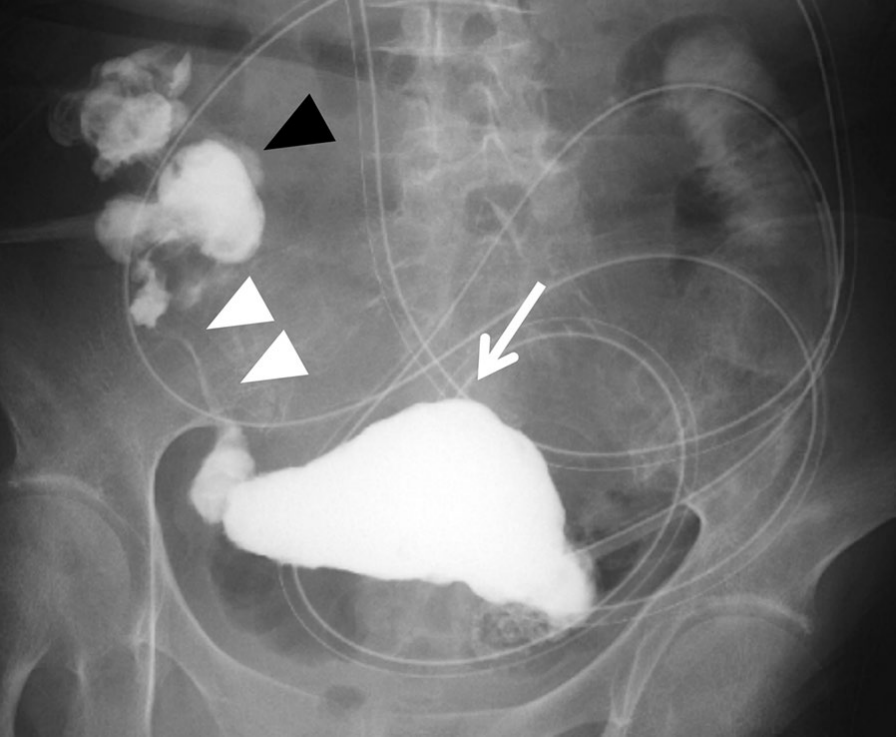
Contrast study from the transnasal decompression tube shows distention (arrow), stenosis (white arrowhead) of the terminal ileum, and normal ascending colon (black arrowhead)

**Fig. 3:**
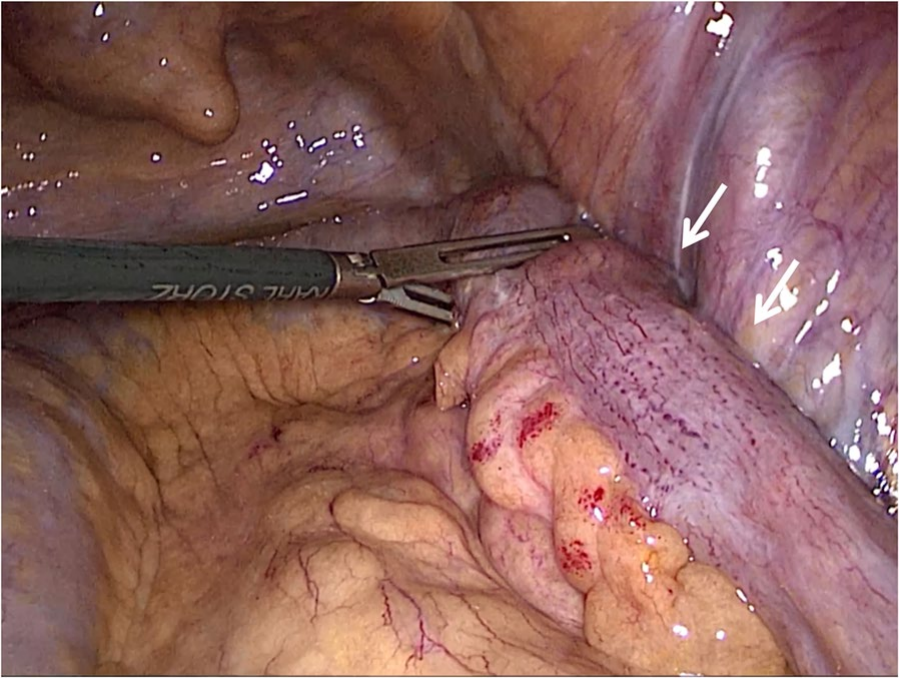
Laparoscopy finding shows the telangiectasia of the terminal ileum serosa and an elastic hard mass-like lesion was palpable with laparoscopic forceps (white arrow)

**Fig. 4:**
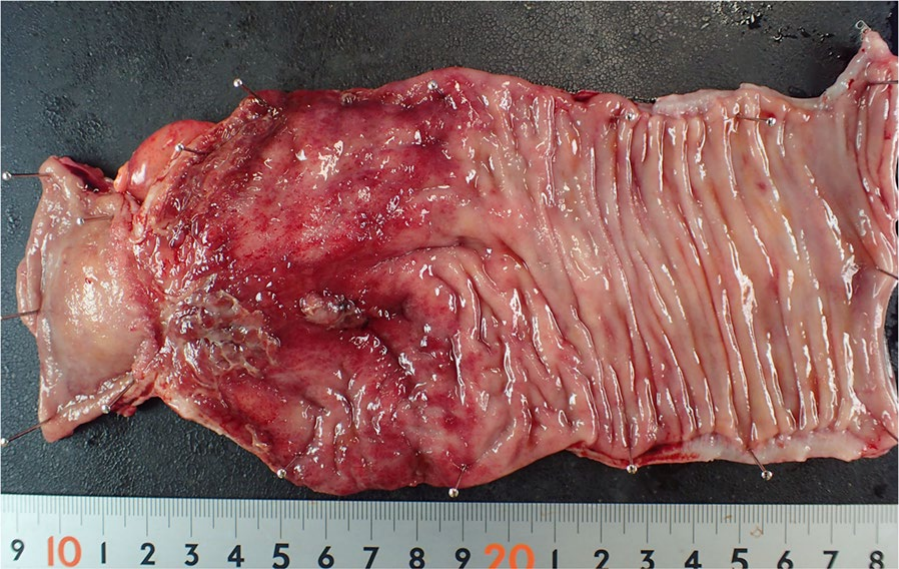
Macroscopic findings show a thickened wall, circumferential ulceration with scarring and redness of the mucosa, which is consistent with the segment of the stricture

**Fig. 5:**
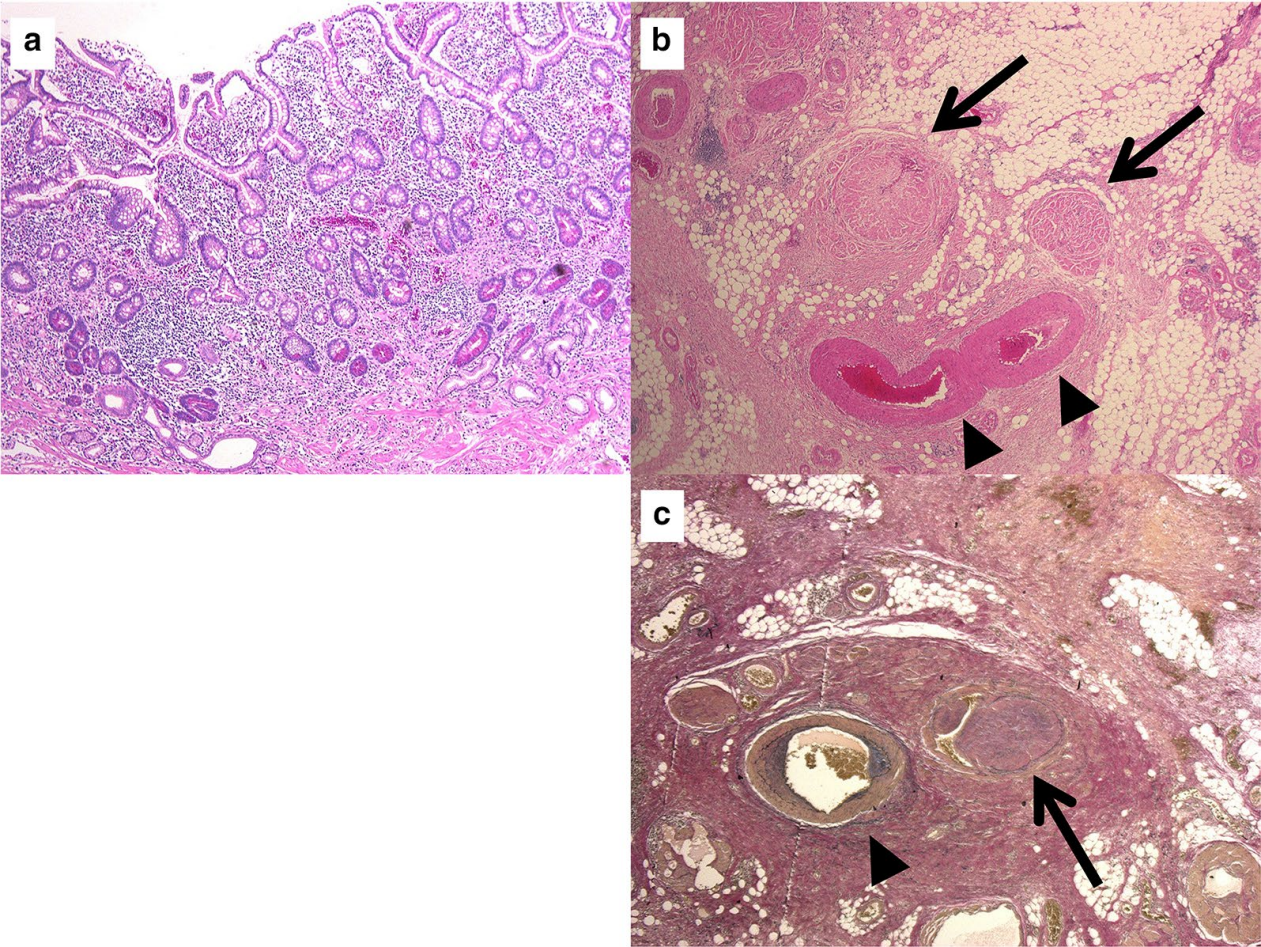
Microscopic findings: Active inflammation and fibrotic change are observed (**a** Hematoxylin and eosin staining, × 40). Thickening of the venous wall is observed (black arrow), and the veins seem more prominent than their accompanying arteries (arrowhead) and have a markedly narrowed lumen (**b** Hematoxylin and eosin staining, × 200). The elastin staining allows hyperplastic veins (arrow) to be distinguished from the normal arteries (arrowhead), which show a bilayer structure (internal and external elastic lamina) (**c** Elastica van Gieson staining, × 200)

**Fig. 6:**
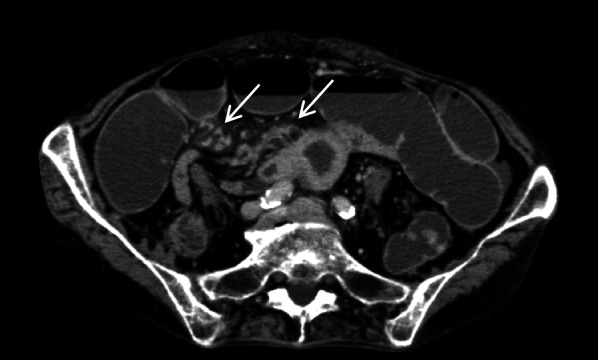
The enlarged and winding peripheral marginal veins are observed on computed tomography (white arrow)

An 81-year-old woman was admitted to our hospital with mild abdominal pain, nausea, and vomiting. She had a history of open appendectomy and laparoscopic cholecystectomy. She had received conservative therapy for small bowel obstruction twice in the previous year. Thus, adhesive intestinal obstruction was suspected at admission. A physical examination showed a slightly distended abdomen with diffuse and mild abdominal tenderness. A hematological examination showed elevated inflammatory markers (white blood cell, 17,000/µl; C-reactive protein, 0.63 mg/dl). Abdominal X-ray showed air fluid level of the small intestine. Contrast-enhanced computed tomography (CECT) demonstrated stenosis and the wall thickness of the terminal ileum and intestinal distention of the proximal small intestine, which was the site that had been affected in the first bowel obstruction event one year previously (Fig. 1). Colonoscopy performed 18 months previously at another hospital showed no abnormalities in the colon or rectum. Based on the above findings, recurrence of the small bowel obstruction at the terminal ileum due to adhesion was suspected. Transnasal decompression was attempted as an initial treatment. X-ray with contrast medium revealed stenosis of the ileum (Fig. 2). We planned to perform laparoscopic surgery to treat small bowel obstruction. During laparoscopic surgery, no adhesion of the small intestine was observed in the abdominal cavity and telangiectasia was pointed out on the serosa of the terminal ileum. This lesion was palpable as an elastic hard mass when gently grasping it with laparoscopic forceps. (Fig. 3). Threfore, partial resection of the intestine was performed. Macroscopic observation of the excised specimen revealed a thickened wall and circumferential ulceration with scarring in the stenotic segment (Fig. 4). A histopathological examination showed active inflammation and fibrotic change with lymphocytic and plasmacytic infiltration in the intestinal mucosa, lamina propria and subserosa (Fig. 5a). In addition, a thickened venous wall, stenosis, and obstruction of the venous lumen in the subserosa were observed (Fig. 5b). Elastica van Gieson staining confirmed the presence of elastic fiber at the site of the thickened venous intima (Fig. 5c). The wall structure of the veins was similar to that of the arteries, while there were no findings of arteritis. Phlebitis and phlebosclerosis were not observed. These findings were consistent with IMHMV. Retrospectively, we recognized that preoperative CECT showed dilation and winding change of the peripheral veins in the ileocecal region with edematous wall thickening, which supported the diagnosis of IMHMV (Fig. 6). The patient had no postoperative complications and left the hospital on the seventh postoperative day. At two years and 8 months after the operation, the patient was well without any additional medication.

## Discussion

IMHMV is a rare disease that was first described by Genta and Haggitt in 1991 [3]. It is characterized by myointimal proliferative changes that narrow the lumina of mesenteric veins in combination with ischemic injury and ulcers of the intestinal tract without thrombosis [3]. IMHMV tends to affect young healthy males, and to occur at the rectosigmoid colon. [4]. A search of the PubMed database for articles published until April 2020 with the key words “idiopathic myointimal hyperplasia of mesenteric veins” or “IMHMV”, yielded 37 cases of IMHMV, with the rectosigmoid colon affected in most cases (83.8%; 31/37) [1, 4–9]. In contrast, the small intestine was affected in only five cases (13.5%; 5/37) [4, 10–13]. Table 1 shows a summary of five previous cases with small intestinal IMHMV and our present case. Regarding colonic IMHMV, the clinical symptoms included persistent abdominal pain, tenesmus and hematochezia, and colonoscopic and CECT findings included stricture, ulcers, and mucosal erythema with intestinal wall thickening, which are similar to the findings of IBD or ischemic colitis [1, 4–9]. In contrast, small bowel IMHMV is more common in females than in males. Additionally, small intestinal IMHMV was associated with various clinical symptoms. Interestingly, previous reports presented that 40% of patients with small intestinal IMHMV were diagnosed with small bowel obstruction before surgery. In addition, hematochezia appeared in only one of five patients with small intestinal IMHMV [4, 10–13]. Our case was also diagnosed preoperatively as small bowel obstruction with symptoms such as abdominal pain and vomiting without hematochezia, and the imaging examinations revealed that a portion of the ileum was obstructed. Considering these findings, the symptoms of IMHMV of small intestine might be quite different from those of IMHMV of the rectosigmoid colon. One possible explanation for this difference might be the smaller luminal diameter of the ileum in comparison to the colon. Accordingly, the inflammation of IMHMV might more strongly influence the caliber change of the small intestine, resulting in bowel obstruction.

**Table 1:** Summary of previous reports on idiopathic myointimal hyperplasia of the mesenteric veins involving the small intestine

Year	Authors	Age	Sex	Affected site	Clinical impression	Time to surgery	Follow up
1998	Bryant et al. [10]	42	F	Jejunum	N.D.	N.D.	N.D.
2012	Lanitis et al. [11]	81	M	Ileum	Subobstruction and ascites	6 months	N.D.
2015	Laskaratos et al. [12]	62	F	Ileum	Deep ulceration and perforation	N.D.	N.D.
2016	Song et al. [4]	59	M	Ileum to sigmoid colon	Exacerbation of Crohn’s disease	30 years	N.D.
2017	Guadagno et al. [13]	59	F	Ileum	Subobstruction due to IBD	6 months	3 years
2019	Our case	81	F	Ileum	Adhesive intestinal obstruction	1 year	2 years 8 months

*N. D.* not documented, *IBD* Inflammatory bowel disease

In previous reports, no cases of small intestinal IMHMV were diagnosed without bowel resection. The preoperative diagnosis of IMHMV is still difficult because there are few specific image findings. However, a precise preoperative diagnosis of IMHMV is important, since IMHMV can be completely cured by resection of the affected area [4], which is an important difference from IBD. Yun et al. reported that the finding of occlusion of the distal inferior mesenteric vein with peripheral venous ectasia on inferior mesenteric angiography could be useful for the preoperative diagnosis of IMHMV of the colon. In addition, they retrospectively reviewed CECT using a 3D workstation, and confirmed aneurysm-like change of the pericolic veins corresponding to the site of venous ectasia on angiography, indicating that arteriovenous fistula was the etiology of IMHMV as previously reported [14, 15]. We retrospectively reviewed the preoperative CECT images and found dilation and winding change of the peripheral veins in the ileocecal region with edematous wall thickening. As a result of these imaging findings, CECT might be useful for the accurate diagnosis of IMHMV. However, this disease might still be difficult to diagnose and may be relatively rare. Thus, an intraoperative pathological examination should be considered to rule out other diseases, including malignancy.

Enterocolic lymphocytic phlebitis (ELP), known as mesenteric inflammatory veno-occlusive disease (MIVOD), is a venogenic intestinal disease similar to IMHMV in terms of its association with intimal hyperplasia of the mesenteric veins [16–18]. However, unlike IMHMV, ELP tends to occur in middle-aged men and women, and in most cases, the affected sites are the right colon and terminal ileum [1]. Histologically, the major histological difference between IMHMV and ELP is the evidence of vasculitis [17, 18]. Although the clinical presentation at the affected site and the sex of the patient in this case suggested the possibility of ELP, microscopy did not show vasculitis. On the other hand, Nakaya et al. previously pointed out that venous damage observed in IMHMV cases of the small intestine might be due to chronic ELP. Chronic ELP had relatively mild lymphocyte infiltration and a chronic disease course, similar to IMHMV, as was seen in this case [16]. Thus, there is a possibility that IMHMV and chronic ELP might belong to same disease spectrum or that IMHMV of small intestine might occur in the course of chronic ELP. The accumulation of further cases is necessary for future discussion.

## Conclusion

We described a rare case of IMHMV of the ileum with intestinal obstruction.

## Data Availability

Not applicable.

## Abbreviations

IMHMV: Idiopathic myointimal hyperplasia of mesenteric veins
IBD: Inflammatory bowel disease
CECT: Contrast-enhanced computed tomography
ELP: Enterocolic lymphocytic phlebitis
MIVOD: Mesenteric inflammatory veno-occlusive disease
